# Metallocene Antimalarials: The Continuing Quest

**DOI:** 10.1155/2008/495123

**Published:** 2007-10-28

**Authors:** Margaret A. L. Blackie, Kelly Chibale

**Affiliations:** ^1^Department of Chemistry, University of Cape Town, Rondebosch 7701, South Africa; ^2^Institute of Infectious Disease and Molecular Medicine, UCT Faculty Of Health Sciences, University of Cape Town, Observatory 7925, South Africa

## Abstract

Over the last decade, a significant body of research has been developed around the inclusion of a metallocene moiety into known antimalarial compounds. Ferroquine is the most successful of these compounds. Herein, we describe our contribution to metallocene antimalarials. Our approach has sought to introduce diversity sites in the side chain of ferroquine in order to develop a series of ferroquine derivatives. The replacement of the ferrocenyl moiety with ruthenocene has given rise to ruthenoquine and a modest series of analogues. The reaction of ferroquine and selected analogues with Au(PP${\text{h}}_{3}$)${\text{NO}}_{3}$, Au(${\text{C}}_{6}{\text{F}}_{5}$)(tht), and [Rh(COD)${\text{Cl}}_{2}$] has resulted in a series of heterobimetallic derivatives. In all cases, compounds have been evaluated for in vitro antiplasmodial activity in both chloroquine-sensitive and chloroquine-resistant strains of *Plasmodium falciparum.* Preliminary structure-activity relationships have been delineated.

## 1. INTRODUCTION

The ongoing battle against malaria is far from over. In the 1950s and 1960s, there was a massive drive to try and eradicate malaria worldwide following the successful eradication of the disease in the United States. The population at risk from malaria was reduced to 10 %. However, the banning of dicophane (DDT) and the concurrent emergence of chloroquine resistance led to the collapse of this campaign. A quarter of a century later, over 300 million clinical cases of malaria occur annually and over 40 % of the world’s population is at risk of contracting the disease. Of these cases, over one million will prove fatal [1]. The greatest tragedy of malaria is that 90 % of fatalities occur in sub-Saharan Africa, and the overwhelming majority of those fatalities are children under the age of 5. Malaria is still essentially a tropical disease, but continues to claim the title of one of the leading killers among infectious diseases. The importance of developing new antiplasmodial drugs cannot be overemphasised. The Roll Back Malaria campaign which began in 1998 has yet to show a decrease in malaria mortality rates [2].

Over the last decade, there has been an increasing interest in metal-containing antiplasmodials. This trend was in part initiated by the successes of metal-containing antitumour drugs such as cisplatin. In the 1980's and 1990’s, interest in coordination complexes of known chemotherapeutic agents began to emerge [3, 4]. It was not long before coordination complexes of chloroquine were synthesised and evaluated for efficacy against both chloroquine-sensitive and chloroquine-resistant strains of *P. falciparum* [5–7]. It is not surprising that antiplasmodial efficacy proved to be somewhat dependent on both metal and ligand.

The incorporation of a metallocene into compounds with medicinal application was rare prior to the 1980's. The low cytotoxicity of ferrocene, coupled with its lipophilicity (logP_octanol/water_ = 3.28) and its electrochemical behaviour (redox potential of the ferrocene/ferrocenium couple, E_0_ = +0.400 V versus SCE (Saturated Calomel Electrode)), suggested that this compound could yield interesting results if incorporated into a known drug [8]. Indeed there are several reported successes of increased efficacy of ferrocenyl analogues of known drugs. Ferrocifen, the ferrocenyl analogue of tamoxifen, has shown antiproliferation effects in both hormone-dependent and hormone-independent tumours. Tamoxifen and other established chemotherapeutic agents fail to exhibit such antiproliferative effects in both hormone-dependent and hormone-independent cell lines [9]. 

 Ferroquine and other ferrocenyl chloroquine analogues have been shown to be efficacious in vitro in both chloroquine-sensitive and chloroquine-resistant *P. falciparum* across a variety of strains [10–12].

The purpose of this paper is to draw together some of the research carried out within our laboratories in the field of metal-containing chloroquine derivatives. This research, together with work published recently by other researchers, will hopefully shed some light on the role of ferrocene in ferroquine and related compounds.

## 2. FERROQUINE ANALOGUES AND DERIVATIVES

Brocard and colleagues have developed a number of ferrocene-containing chloroquine analogues [10]. Ferrocene was incorporated as an integral part of the side chain; as a terminal component of the side chain; and bonded through the quinoline nitrogen [11–13]. They have shown that incorporation of a ferrocenyl moiety as an integral part of the side chain of chloroquine between the two nitrogens had superior efficacy to other analogues in which the moiety was terminal on the side chain or bonded to the quinoline nitrogen. Some analogues of the compound were produced bearing different alkyl groups on the terminal tertiary nitrogen. They established that the dimethylamino terminal group was superior in efficacy [10].

Building on this foundation, we decided to explore the possibility of developing other analogues of ferroquine in which a reactive secondary amine group between the quinoline and ferrocenyl moieties would serve as a site for introducing further chemical diversity. We reasoned that this would facilitate the exploration of structure-activity relationships. The role of the length of the methylene spacer between the two nitrogens in chloroquine analogues has been shown to have an influence on efficacy in chloroquine-resistant strains of *P. falciparum* [14]. Krogstad and coworkers have shown that aminoquinolines with short (2-3 carbons) and long (10–12 carbons) methylene side chains are equipotent against chloroquine-sensitive, chloroquine-resistant, and multidrug-resistant strains of *P. falciparum*. Whilst aminoquinolines with side chains of intermediate length (4–8 carbons) showed efficacy against chloroquine-sensitive strains of *P. falciparum*, they showed a significant decrease in efficacy against chloroquine-resistant strains of *P. falciparum* [15]. For this reason, we decided to explore the influence of chain length on ferroquine analogues. The parallel series of urea derivatives was also synthesised and evaluated at the same time. The introduction of structural diversity via the urea functionality is a well-established strategy in the development of new biologically active compounds [16].

**Scheme 1:**
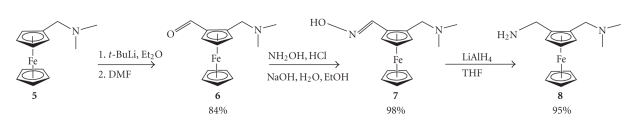
Synthesis of [(*N,N*-dimethylamino)methyl] ferrocenecarboxaldehyde (**6**), and 2-[(*N,N*-dimethylamino)methyl] ferrocenemethylamine (**8**).

**Scheme 2:**
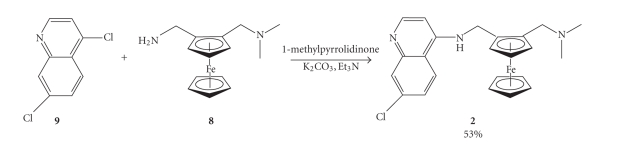
Synthesis of ferroquine (**2**).

**Scheme 3:**
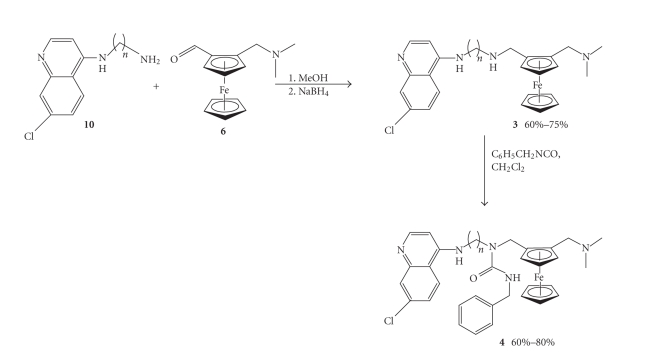
Synthesis of ferroquine analogues (**3a–d**) and urea derivatives (**4a–d**).

Synthesis of the ferroquine-type compounds was achieved via the synthetic strategy shown in Schemes 1–3. The reaction of **5** with *n*-butyllithium gave the desired product in a reasonable yield (70%) [10]. However, the use of *tert*-butyllithium had the effect of reducing the reaction time and improving the yield [17]. The presence of the dimethylaminomethyl substituent on the ferrocene results in a high regioselectivity for the 1,2-disubstituted product **6**.

Ferroquine (**2**) was synthesised from 2-[(*N,N*-dimethylamino)methyl] ferrocenemethylamine (**8**) and 4,7-di- chloroquinoline. The published procedure uses dichloromethane and brine for the workup, in order to extract the product from 1-methylpyrrolidinone [10]. We favoured the use of ethyl acetate rather than dichloromethane as it was more time efficient. This had little impact on the moderate yield of the reaction (53%).

Synthesis of compound **10** was achieved from 4,7-dichloroquinoline and the appropriate 1,n-alkyldiamine. The two starting materials were simply reacted together in the melt to form the product [18]. Compound **10** was then reacted with the ferrocenecarboxaldehyde (**6**) in methanol, to form the imine (Schiff base) which was then reduced in situ with sodium borohydride to deliver compound 3 which was in turn reacted with phenyl isocyanate in dichloromethane at room temperature (see Scheme 3).

Consistent with previous observations [15, 16], the length of the methylene spacer was found to have an influence on antiplasmodial activity. In the chloroquine-sensitive D10 strain, the longer the methylene spacer, the lower the efficacy. A distinguishable pattern was not quite so clear in the chloroquine-resistant K1 strain, but the methylene spacer length appeared to have an impact on efficacy with compound **3b** (3-carbon spacer) showing the greatest efficacy [19]. For the urea derivatives, the chain length made no significant difference to efficacy in D10, but a decrease in efficacy with an increase in the length of the methylene spacer was observed in the K1 strain [19].

**Figure 2:**
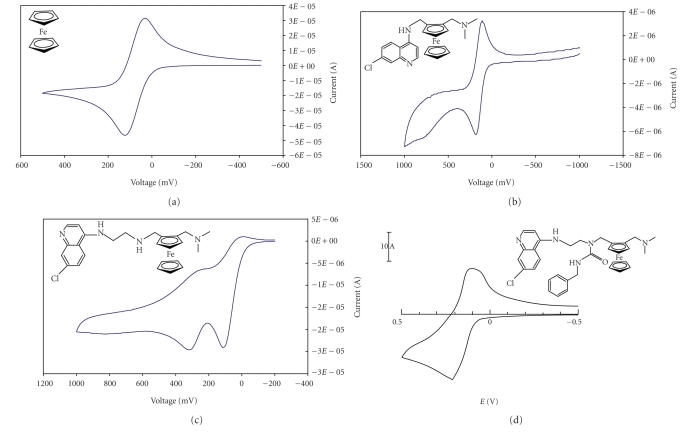
Cyclic voltammograms of selected ferrocene, ferroquine, **3a**, and **4a**.

Given the distinctive redox chemistry of ferrocene, we decided to establish the electrochemical behaviour of the analogues we had synthesised (see Table 2). It was postulated that the half-wave potential *E_1/2_* or *ΔE* value could provide a cheap and quick method of screening for potential biological activity if this could be correlated to the in vitro antiplasmodial activity. Unfortunately, no discernable trend was observed relating either of these values with in vitro efficacy in either strain of *P. falciparum*. However, it was noted that the presence of the reactive secondary amine centre had a marked effect on the redox chemistry of the ferrocenyl moiety. Ferroquine exhibits a fully reversible one-electron oxidation. The curve is similar to that of ferrocene (see Figure 2) although the ferrocenyl moiety in ferroquine is significantly more difficult to oxidise (*E*_1/2_ for FQ = 147; *E*_1/2_ for Fc = 79).

Compounds **3a–d** showed, at best, a quasireversible oxidation. The cathodic peak current was significantly smaller than the anodic peak current (see Figure 2). The ease of reversibility of oxidation increases with an increase in carbon chain length, as demonstrated by the decrease in Δ*E*_p_ values (see Table 2). A fully reversible one-electron oxidation requires a peak separation, Δ*E*_p_, of 70–90 mV. In compounds **4a–d**, the reversibility of the one-electron oxidation event in the ferrocenyl moiety was restored. As with ferroquine, these compounds are significantly more difficult to oxidise than ferrocene, as demonstrated by the increase in half-wave potential *E*_1/2_ values (see Table 2).

## 3. RUTHENOQUINE ANALOGUES

**Figure 3:**
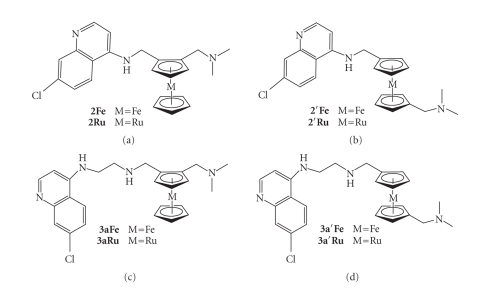
Ferrocene and ruthenocene analogues of ferroquine.

The chemistry of ferrocene is similar to the chemistry of ruthenocene. In order to begin to probe the role of ferrocene in ferroquine derivatives, we reasoned that it would be of interest to synthesise the ruthenocene analogues of selected derivatives (see Figure 3). The antiproliferative properties of ferrocifen and its ruthenocene analogue have proven quite different. This difference in efficacy has been attributed to the stability of the ferrocenium ion relative to the ruthenocenium ion. The presence of the ferrocenium ion allows for a change in chemical reactivity at another point in the molecule as a result of the highly conjugated system present in the ferrocifen molecule. The instability of the ruthenocenium ion does not allow for this change in chemical reactivity [9]. It is clear that a similar through-bond effect is not available in ferroquine or ruthenoquine as there is no conjugation accessible to the metallocene moiety in these compounds. However, it was thought that some insight into the role of ferrocene could be gained by examining the biological activity of the ruthenocene analogues.

**Scheme 4:**
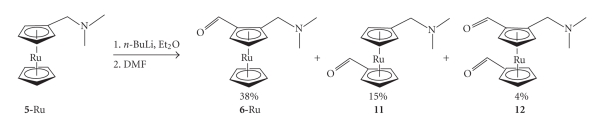


It was envisioned that a similar synthetic strategy could be employed to synthesise the compounds **2Ru** and **3aRu**, as had been used for the ferrocene analogues and described in Schemes 1–3. However, the ruthenocene system proved more complicated [20]. Firstly, (*N,N*-dimethylaminomethyl) ruthenocene is not commercially available and had to be synthesised using Eschenmoser's salt ([CH_2_=NMe_2_]I) [21]. Secondly, in the ferrocenyl system, high regioselectivity for the 1,2-disubstituted product **6** is observed. In the ruthenocenyl system, when using *n*-butyllithium to achieve deprotonation, significant quantities of the 1,1′-monoaldehyde **11**, and the dialdehyde **12** were formed (see Scheme 4). Isolation of **11** led to the synthesis of isoruthenoquine, **2′Ru**, and **3a′Ru**. It was later discovered that the use of *tert*-butyllithium affords **6-Ru** exclusively. However, by this stage, **2′Ru** and **3a′Ru** had already been synthesised and found to show antiplasmodial activity [21]. In order to create a better comparison of the efficacies of the ferrocene and ruthenocene compounds, the 1,1′-ferrocenyl derivatives were also synthesised. The synthesis of the 1,1′-ferrocene compounds required a fairly lengthy synthetic procedure which is discussed in full elsewhere [22].

All compounds tested exhibited a good efficacy in both chloroquine-sensitive and chloroquine-resistant strains. Consistent with previous findings, the addition of the metallocene was advantageous in overcoming chloroquine resistance. These results indicate that the 1,2-disubstituted compounds (**2Fe**, **2Ru**, **3aFe**, and **3aRu**) are more efficacious than the 1,1′-disubstituted analogues (**2′Fe**, **2′Ru**, **3a′Fe**, and **3a′Ru**) in the chloroquine-resistant K1 strain. It has not been established why this trend should be observed, but the crystal structures of these compounds clearly show that there is a significant increase in distance between the 4-aminoquinoline nitrogen and the terminal nitrogen when moving from the 1,2- to 1,1′-disubstituted product [21, 23]. It has been previously established that this distance is significant in the observed efficacy of 4-aminoquinolines [15, 16]. It is noteworthy that barring compounds **3a′Fe** and **3a′Ru**, there is no significant difference in efficacy between ferrocenyl and ruthenocenyl analogues. This may suggest that the difference in redox chemistry and chemical reactivity of these moieties is not a factor in the efficacy of these compounds. The observed efficacy may be associated with the lipophilicity or physical bulk of the metallocene group.

**Figure 4:**
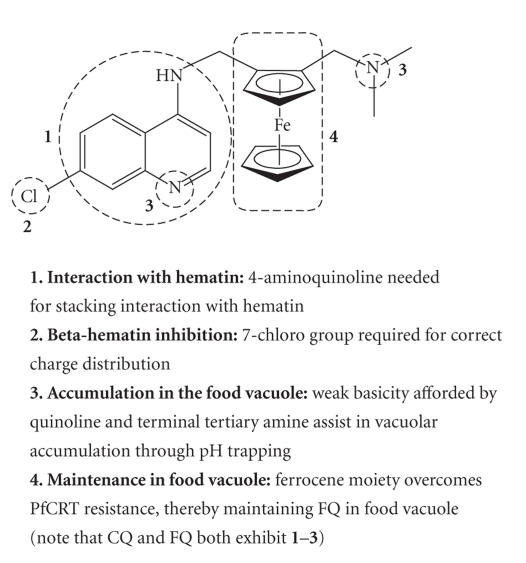
Proposed structure-activity relationships for ferroquine [23].

Comparative studies on the behaviour of chloroquine and ferroquine have led to the conclusion that the effect of shape, volume, lipophilicity, basicity, and electronic profiles of the ferrocenyl moiety leads to a modification of the pharmacological behaviour of the analogue [23]. It has yet to be established which of these factors is most significant. In chloroquine-resistant strains of *P. falciparum*, a transporter protein, *P. falciparum* chloroquine resistance transporter (PfCRT), has been identified which allows the efflux of chloroquine from the food vacuole. It has been noted that ferroquine resistance cannot be induced under drug pressure in the W2 strain of *P. falciparum*. This has led to the conclusion that the bulky lipophilic ferrocene moiety overcomes PfRCT resistance, thereby maintaining ferroquine in the food vacuole. In addition, ferroquine retains all the features that have been identified as necessary in the structure of chloroquine (see Figure 4) [23]. The similarity of behaviour of the ruthenoquine and ferroquine molecules opens an interesting avenue of research. Ruthenium is known to be a good contrasting agent in electron microscopy [24]. It is reasonable, in the absence of data to the contrary, to postulate that the mechanism of action of ruthenoquine and ferroquine is similar. The sites of accumulation of ruthenoquine can be established easily using scanning electron microscopy. In mice infected with *Plasmodium berghei* N and treated with ruthenoquine, the drug has been found to accumulate close to the malaria pigment and in the parasitic membrane. Chloroquine exhibits no such accumulation in the parasitic membrane [25]. It is possible that ferroquine exhibits a similar pattern of accumulation to ruthenoquine, although this has yet to be established. The reason for this accumulation of ruthenoquine in the membrane has not been ascertained, but it may well be associated with the increase in lipophilicity afforded by the metallocene moiety.

## 4. COORDINATION COMPLEXES

A number of transition metals have been used to form coordination complexes of chloroquine. These coordination complexes have been shown to exhibit improved efficacy in both chloroquine-sensitive and chloroquine-resistant strains of *P. falciparum* compared to chloroquine [6,7,26]. The [Ru(Cl)_2_(CQ)]_2_ dimeric complex showed considerably better in vivo efficacy than was expected on the basis of the in vitro results [6]. The same study reports that both [Rh(COD)Cl]_2_ and RuCl_2_(DMSO)_4_ failed to exhibit any antiplasmodial activity. As the metal complexes of chloroquine showed improved efficacy against both chloroquine-sensitive and chloroquine-resistant strains of the parasite, it was concluded that the coordination of a metal to the quinoline nitrogen enhanced the efficacy of chloroquine in vitro. However, a decade after the advent of these and other chloroquine-metal coordination complexes, the mode of action of these complexes is still poorly understood. It has not been established whether these complexes reach the site of action intact or not. Furthermore, it has not been established whether the inhibition of *β*-haematin formation is possible with the presence of a coordinated metal. This question is relevant as it has been established that the 7-chloro-4-aminoquinoline structure is crucial for this mode of action in chloroquine [27].

**Figure 5:**
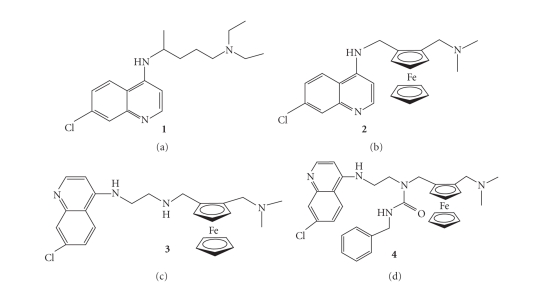
Ligands for coordination.

Given that the ferroquine derivatives could give rise to an analogous series of ferroquine coordination complexes, we thought it would be interesting to examine the effect of coordination of a second metal. To our knowledge, no such heterobimetallic coordination complexes of chloroquine had been synthesised and tested for antiplasmodial efficacy [28]. Four ligands (**1–4**), in Figure 5, were selected for preliminary comparative studies.

The results shown in Table 4 indicate that the chloroquine complexes [Au(CQ)(PPh_3_)]NO_3_, [Au(C_6_F_5_)(CQ)], and [Rh(Cl)(COD)(CQ)] all show comparable efficacy to chloroquine in the chloroquine-sensitive strain. In the chloroquine-resistant strain, these complexes exhibit an efficacy 7 to 9 times better than chloroquine. However, it is noteworthy that efficacy of these compounds dropped up to three times in moving from sensitive to resistant strain suggesting that some cross-resistance may occur. Unfortunately, the metal complexes of the ferrocenyl-4-aminoquinolines did not perform so well. At best, addition of the second metal had little effect on the efficacy of the parent ligand (as in pentafluorophenyl gold complexes of **L2**, **L3**, and **L4**), whilst at worst, there appeared to be a significant antagonistic effect (as in the cyclooctadiene rhodium complexes of **L3** and **L4**). Whilst the reason for this antagonistic effect was not explored, it was noted that the presence of the second metal made the ferrocenyl moiety far more difficult to oxidise. This was evidenced by the increase in half-wave potential, *E*_1/2_, in the complexes of **L2** and **L4** when compared with the free ligand. In the case of **L3**, where no fully reversible one-electron oxidation was observed, the significant increase in cathodic peak potential suggests that the ferrocenyl moiety is more difficult to oxidise. Once again we found no discernible correlation between redox activity and antiplasmodial efficacy.

## 5. DISCUSSION

The ferrocenyl moiety has several characteristics which make it a good addition to known drug molecules. Its lipophilicity, electron density, relative thermal and chemical stability, and interesting redox behaviour are all favourable in this respect. We have shown that altering the structure of the ferroquine analogue can have a significant effect on the redox behaviour of the ferrocenyl moiety. However, there is no discernible correlation between the differences in redox behaviour, whether ease of oxidation or reversibility of oxidation, and the efficacy of a compound against either chloroquine-sensitive or chloroquine-resistant strain of *P. falciparum*. It is worth noting that ferrocene and ruthenocene have quite different redox chemistry. The ruthenocenyl analogue of the antitumour agent ferrocifen has been shown to have a significantly different range of efficacy which is attributed principally to this difference in redox chemistry and the effect on the highly conjugated molecule to which the metallocene is bonded. No such difference in antiplasmodial efficacy is observed here between ferrocenyl and ruthenocenyl analogues of ferroquine. This, together with our cyclic voltammetric studies and the lack of correlation with antiplasmodial efficacy, suggests that the redox behaviour of the metallocene is not a significant factor in the efficacy of these compounds. Some study of the redox chemistry of the ruthenoquine analogues would be useful to determine the accuracy of this supposition.

The incorporation of a ferrocenyl moiety into chloroquine has yielded fruitful results in terms of overcoming chloroquine resistance. Ferroquine continues to undergo testing to ascertain its suitability as a candidate for full-scale human clinical trials [8]. However, the results of incorporation of a ferrocenyl moiety into other known antimalarial drugs such as mefloquine, quinine [29], or artemisinin [30] have proved far less rewarding. This raises a question as to whether there is any inherent antiplasmodial toxicity associated with the ferrocenyl moiety. It may be that the changes in lipophilicity and *pK_a_* values and other physicochemical effects of the incorporation of the ferrocenyl moiety into chloroquine are the primary factor in the enhanced efficacy of ferroquine. This may also explain the antagonistic effects exhibited by the heterobimetallic complexes of ferroquine and its analogues. The presence of the complexed metal and associated ligands may have adverse effects on the influence of the ferrocenyl moiety.

The role of the ferrocenyl moiety in the ability of ferroquine to overcome chloroquine resistance has still not been fully determined, although its role in overcoming PfCRT resistance seems supported by experimental evidence [24]. The continuing biological success of ferroquine means that this avenue of research must remain open and active. If we are to overcome the problem of malaria in sub-Saharan Africa, a potent, cheap alternative to chloroquine must be found.

## Figures and Tables

**Figure 1:**
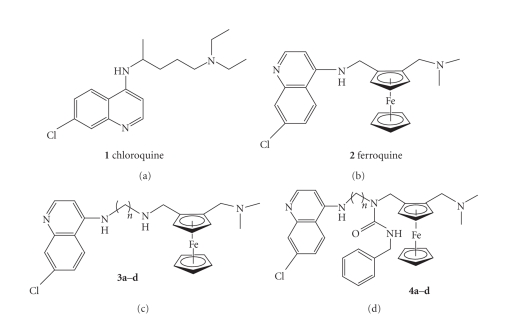
Structure of ferroquine analogues.

**Table 1:**
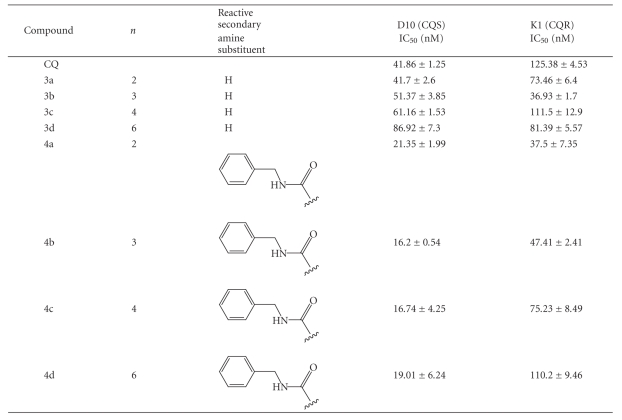
In vitro antiplasmodial activities of ferroquine analogues.

**Table 2:**
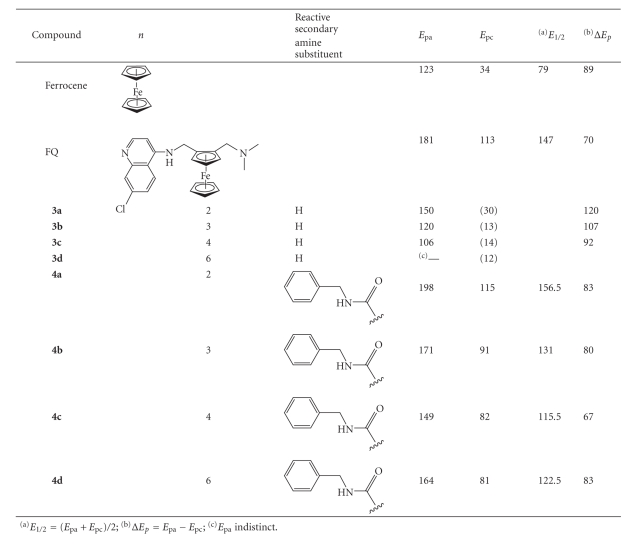
Cyclic voltametry measurements on antimalarial ferroquine analogues.

**Table 3:** Results of in vitro antimalarial tests conducted on the chloroquine-sensitive (D10) and chloroquine-resistant (K1) strains of *P. falciparum*.

Compound	D10	K1
	IC_50_/nM	IC_50_/nM
CQ·2H_3_PO_4_	23	352
**2Fe**	18	14
**2Ru**	19	13
**2′Fe**	19	49
**2′Ru**	25	29
**3aFe**	33	37
**3aRu**	20	20
**3a′Fe**	16	65
**3a′Ru**	34	127

**Table 4:** In vitro activity of coordination complexes and the free 4-aminoquinoline ligands in chloroquine-sensitive (D10) and chloroquine-resistant (K1) strains of *P. falciparum.*

Compound	D10 (CQ sensitive)	K1 (CQ resistant)
	IC_50_(nM)	IC_50_ (nM)
**L1**	37.10	570.0
[Au(**L1**)(PPh_3_)]NO_3_	21.09	61.53
[Au(C_6_F_5_)(**L1**)]	18.07	61.09
[Rh(Cl)(COD)(**L1**)]	21.50	81.31

L2	16.30	4.96
[Au(**L2**)(PPh_3_)]NO_3_	10.50	5.68
[Au(C_6_F_5_)(**L2**)]	10.08	3.96
[Rh(Cl)(COD)(**L2**)]	15.80	10.55

L3	11.30	12.56
[Au(**L3**)(PPh_3_)]NO_3_	33.27	33.89
[Au(C_6_F_5_)(**L3**)]	13.70	14.65
[Rh(Cl)(COD)(**L3**)]	202.00	597.59

L4	16.54	15.54
[Au(**L4**)(PPh_3_)]NO_3_	30.32	31.09
[Au(C_6_F_5_)(**L4**)]	23.36	12.92
[Rh(Cl)(COD)(**L4**)]	56.10	47.70

**Table 5:** Cyclic voltametry of gold triphenylphosphine complexes and the parent ligands.

Compound	^(a)^ *E*_pa_(mV)	^(b)^ *E*_pa_(mV)	^(c)^ *E*_1/2_(mV)	*E*_pa_-*E*_pc_(mV)
**L2**	181	113	147	70
[Au(**L2**)(PPh_3_)]NO_3_	252	162	207	90

**L3**	150	30	—	120
[Au(**L3**)(PPh_3_)]NO_3_	294	186	—	108

**L4**	198	115	158	83
[Au(**L4**)(PPh_3_)]NO_3_	219	141	180	78

^(a)^anodic potential; ^(b)^cathodic potential; ^(c)^half wave potential (*E*_pa_ + *E*_pc_)/2.
